# Study of Combined Multi-Point Constraint Multi-Scale Modeling Strategy for Ultra-High-Performance Steel Fiber-Reinforced Concrete Structures

**DOI:** 10.3390/ma13235320

**Published:** 2020-11-24

**Authors:** Zuohua Li, Zhihan Peng, Jun Teng

**Affiliations:** 1 Shenzhen Key Lab of Urban & Civil Engineering Disaster Prevention & Reduction, Harbin Institute of Technology, Shenzhen 518055, China; lizuohua@hit.edu.cn (Z.L.); hayespeng@gmail.com (Z.P.); 2 School of Civil and Environment Engineering, Harbin Institute of Technology, Shenzhen 518055, China

**Keywords:** ultra-high-performance steel fiber-reinforced concrete, multiscale finite element modeling, multi-point constraint, multi-scale interface connection, concrete damage plasticity model, ABAQUS

## Abstract

Compared with normal strength concrete (NSC), ultra-high-performance steel fiber-reinforced concrete (UHPFRC) shows superior performance. The concrete damage plasticity (CDP) model in ABAQUS can predict the mechanical properties of UHPFRC components well after calibration. However, the simulation of the whole structure is seriously restricted by the computational capability. In this study, a novel multi-scale modeling strategy for UHPFRC structure was proposed, which used a calibrated CDP model. A novel combined multi-point constraint (CMPC) was established by the simultaneous equations of displacement coordination and energy balance in different degrees of freedom of interface nodes. The advantage is to eliminate the problem of the tangential over-constraint of displacement coordination equation at the interface and to avoid stress iteration of the energy balance equation in the plastic stage. The expressions of CMPC equations of typical multi-scale interface connection were derived. The multi-scale models of UHPFRC components under several load cases were established. The results show that the proposed strategy can well predict the strain distribution and damage distribution of UHPFRC while significantly reducing the number of model elements and improving the computational efficiency. This study provides an accurate and efficient finite element modeling strategy for the design and analysis of UHPFRC structures.

## 1. Introduction

Concrete is currently the most widely used building material. Although many structures are built with concrete, the use of normal strength concrete (NSC) still has some limitations, such as low tensile strength and low ductility. Improving the mechanical properties of concrete to obtain higher strength and higher ductility has been widely of concern. Ultra-high-performance steel fiber concrete (UHPFRC) is a new type of fiber concrete, with high strength, fracture toughness, and ductility. Its compressive strength and tensile strength are generally over 150 MPa and 7 MPa, respectively [1,2,3,4,5], and even the tensile strength can reach 15 MPa [6]. Ultra-high-performance concrete has been applied and investigated in many kinds of engineering structures, such as concrete structures [7,8,9], seismic design [10,11], etc. For the material level, numbers of studies have been conducted on the influence of fiber types, fiber orientations, geometric shapes, dosages, and other factors on the mechanical properties of ultra-high-performance concrete [12,13,14,15,16,17,18,19]. Numerous experimental studies have been carried out on high-performance concrete components, which include full-size prestressed beams [20,21,22], reinforced beams [23,24,25,26], columns [27], slabs [28], etc.

Extensive tests are required at the material and structural levels in order to develop standard analytical procedures and design specifications for UHPFRC, which will take a lot of time and cost. Therefore, verifying the concrete material models in the existing finite element software by conducting a limited number of well-formulated tests on the material and structure levels is a way to save time and cost. The verified concrete material model and finite element modeling method can be used to establish extended analysis of various design parameters. In addition, the influences of changes in geometry, load cases, and reinforcement on mechanical properties of UHPFRC can be investigated. The finite element software ABAQUS is equipped with the concrete damage plasticity (CDP) model developed for NSC, which is a mature and reliable tool for predicting the mechanical behavior of NSC [29,30,31]. Compared with NSC, the material properties of UHPFRC have higher tensile strength and ductility, which makes the shape of a material constitutive curve substantially different from NSC. In order for CDP model to be used to simulate UHPFRC, the parameters of CDP model need to be calibrated. Tysmans et al. [32] used CDP model to simulate the behavior of high-performance fiber concrete composites under biaxial tension. Mahmud et al. [33] and Singh et al. [34] calibrated the CDP model through the UHPFRC material test and used the calibrated model to simulate the test results of the UHPFRC beam [24]. It was reported that the calibrated CDP model can accurately and effectively predict the load-displacement curves and plastic damage distributions of UHPFRC components.

Similar to the investigations of NSC, the investigations of UHPFRC need to be developed to the structural level as well as the material and component level. However, it is very expensive to establish a full-scale structural test, which is seriously restricted by the test conditions. When the calibrated CDP model is used to simulate a single UHPFRC component, the reduction of mesh size and the increase of number of elements will significantly increase the calculation time, while larger mesh size will lead to convergence problems [34]. Therefore, it is difficult to use solid elements to simulate all of the UHPFRC of the whole structure. Fortunately, the multi-scale finite element simulation strategy can solve this problem. The simulation strategy uses solid elements to simulate the key parts of the structure that need to be paid attention to, and adopts the macro-scale elements such as truss or beam elements for the other parts. Its advantage is to use limited computing resources to ensure the requirements for simulation accuracy and to improve computational efficiency. So the simulation strategy has been well applied in structural failure analysis, seismic design, optimization of structural system, etc. [35,36,37,38,39]. The key problem of the multi-scale finite element simulation strategy is to establish an accurate interface-coupling constraint relationship, so as to ensure the scientific and reasonable coordination between different scale elements. The multi-point constraint method is based on the relations of displacement coordination [40] or energy balance [41] between macro-scale and micro-scale elements at the interface, and the constraint equations containing the degrees of freedom of nodes of different scale elements are established at the interface [42,43]. However, a single multi-point constraint relation has the limitation that the stress state and deformation of the connection interface appear distorted after the material enters the plastic stage [44].

In order to promote the development of finite element simulation of UHPFRC structure, a novel multi-scale finite element modeling strategy was proposed in this study. A novel combined multi-point constraint (CMPC) based on displacement coordination and energy balance was established, aiming at the problems of the tangential over-constraint and the requirements for nonlinear stress iteration existing in the single multi-point constraint method. The nonlinear constitutive relationship of UHPFRC is considered. The multi-scale models of UHPFRC components under various load cases were established in the finite element software ABAQUS. The comparative analysis results show that the proposed multi-scale modeling strategy can well predict the strain distribution and damage distribution of UHPFRC components while significantly reducing the number of model elements and improving the computational efficiency. This study provides an accurate and efficient finite element modeling strategy for the design and analysis of UHPFRC structure, which can promote the application and development of UHPFRC in the construction industry.

## 2. Multi-Scale Modeling Strategy—Material Models

### 2.1. Calibrated Concrete Damage Plasticity (CDP) Model

The concrete damage plasticity (CDP) model is a concrete material model for NSC in the finite element software ABAQUS. It is a mature and reliable tool for predicting the mechanical behavior of NSC [29,30,31]. In order for CDP model to be used to simulate UHPFRC, the CDP model needs to be calibrated. Some studies have shown that the calibrated CDP model can accurately and effectively predict the mechanics characteristic of UHPFRC. In this study, the stress-strain curve for UHPFRC in compression proposed by Singh et al. [34], modified from Lu et al. [45], is used to calculate the data of compressive behavior in the CDP model. The stress-strain curve of UHPFRC specimen of the uniaxial tension test [34] is used to define the tensile behavior in the CDP model. The curves of compression damage and tension damage in the CDP model are defined according to the studies in [33,46], respectively. The parameters of the CDP model adopted in this study are shown in Table 1.

### 2.2. Validation of the Model

#### 2.2.1. Test Specimens

In this study, the UHPFRC beams named as B25-1 and B25-2 [34] are chosen for the validation analysis. The cross section, spans, loading configuration details and reinforcement detail of the all the beams are given in Table 2, where the tensile reinforcement consisted of 20 mm diameter rebar with a yield strength and ultimate strength of 525 and 625 MPa, respectively. The four point bending test applies the same concentrated load symmetrically at a distance of 250 mm from the middle of the beams, resulting in pure bending stresses between the load points.

#### 2.2.2. Finite Element Analysis (FEA) Model

**Figure 1:**
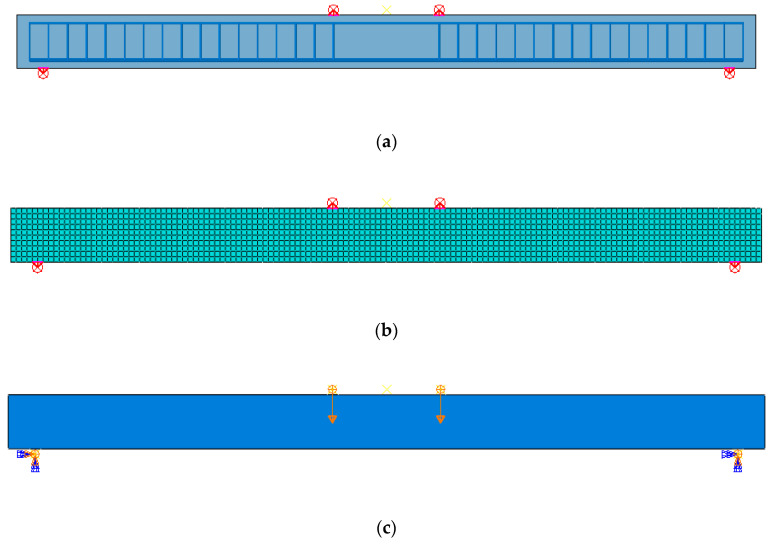
FE model of ultra-high-performance fiber-reinforced concrete (UHPFRC) beam test. (**a**) Reinforcement detail; (**b**) Mesh details; (**c**) Load boundary condition.

According to the design diagram of UHPFRC beam specimen and the design of loading device, the corresponding finite element model of the test was established in ABAQUS with mesh size of 25 mm. The details of the reinforcement, mesh and load boundary condition of the finite element (FE) model are shown in Figure 1. The parameters of the CDP model adopted for the UHPFRC solid elements are shown in Table 1.

#### 2.2.3. Results of FEA Simulation and Test

**Figure 2:**
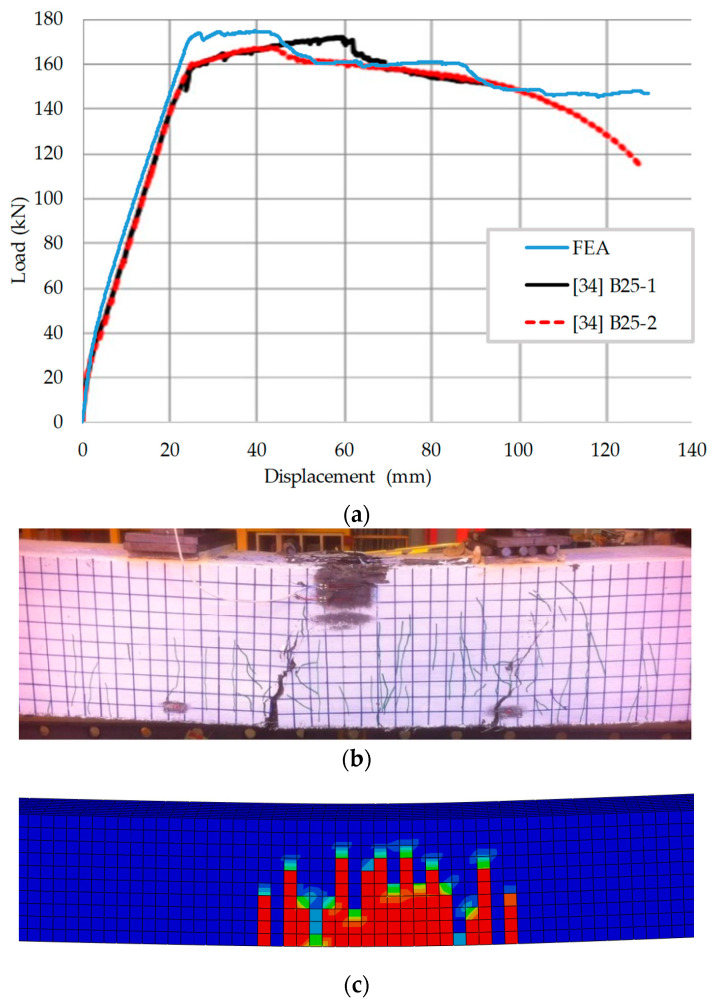
Test results and simulation results of beam test. (**a**) Load-displacement curve; (**b**) failure pattern of the specimen B25-1; (**c**) Tension damage distribution of UHPFRC in the FE model.

The test results and simulation results of the four point bending test of the two UHPFRC specimens are shown in Figure 2, where (a) is the relation curve between the mid-span displacement of the beam and the external load, (b) is the failure pattern of the specimen B25-1, and (c) is the tension damage distribution of UHPFRC in the finite element model. It can be seen that the finite element model can simulate the whole entire load-displacement curve, including the descending section after yielding. The finite element simulation results are in good agreement with the experimental results. The test results and the finite element results of peak load and corresponding displacement of UHPFRC specimens are shown in Table 3. The ultimate load capacity of specimens B25-1 and B25-2 predicted by the finite element model is 3% and 6% higher than the test results, respectively. It can be seen from Figure 2a,b, the damage distribution simulated by the finite element model is similar to the crack distribution of the specimen. Meanwhile, the validity of the parameters selected of the FEA model in this paper is proved so the FEA model with the same parameters can be taken as the standard for the extended study.

## 3. Multi-Scale Modeling Strategy—Interface Connection

### 3.1. Combined Multi-Point Constraint (CMPC) of Multi-Scale Model

#### 3.1.1. Combine Multi-Point Constraint Relations

**Figure 3:**
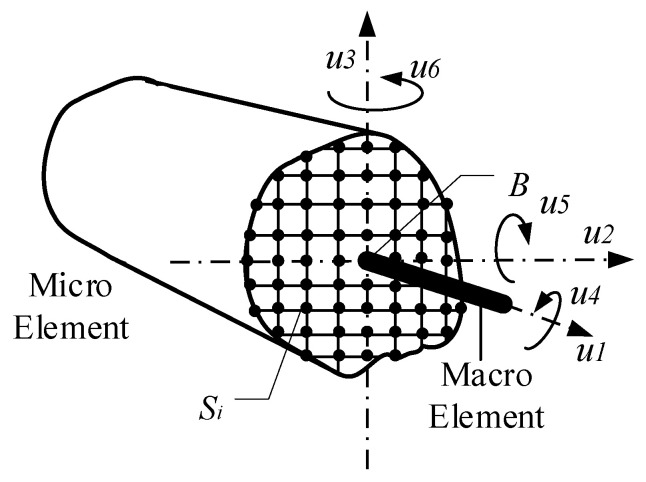
Sketch of the interface connection.

In the multi-scale model, the interface connection of different scale elements can be established by the constraint equations according to the degrees of freedom of interface nodes. The sketch of the interface connection of multi-scale model shown in Figure 3, where *S_i_* (*i* = 1, 2, 3….) signifies micro element nodes with 3 degrees of freedom and *B* signifies macro element node with 6 degrees of freedom.

According to the coupling relation of degrees of freedom of nodes, the unified form of the constraint equations of the multi-scale interface connection is as follows:

*c*(*u*_B_, *u_Si_* ) = *u*_B_ − *Cu_Si_* = 0 (1)

where $u_{B}$ is the displacement vector of macro elements at the interface; $u_{Si}$ is the displacement vector of micro elements at the interface; *C* is the coefficient matrix of interface constraint equations.

The accuracy of multi-scale simulation depends on the rationality of coefficient matrix *C*. If the constraint equations can effectively simulate the actual deformation coordination, a better effect of the coupling can be obtained.

The solution of multi-point constraint equations is usually based on the single constraint relation such as displacement coordination [40] or energy balance [41]. For the multi-scale simulation of UHPFRC structure, due to the nonlinear characteristics of the interface stress and deformation relation in the plastic stage, a single multi-point constraint method will lead to the over-constraint in tangential direction and the requirement of stress iteration in plastic stage. Therefore, the combined multi-point constraint (CMPC) is established in this study through the simultaneous equations of displacement coordination and energy balance. The equations form are as follows:

(2) $$\left\{ \begin{array}{l} u_{1}{}_{Si}-f_{1i}(u_{1}{}_{B},u_{5}{}_{B},u_{6B},b_{i},h_{i})=0\\ F_{2}u_{2B}=\int_{A}\sigma_{2i,F2}u_{2Si}dA\\ F_{3}u_{3B}=\int_{A}\sigma_{3i,F3}u_{3Si}dA\\ F_{4}u_{4B}=\int_{A}(\sigma_{2i,F4}u_{2Si}+\sigma_{3i,F4}u_{3Si})dA \end{array}\right.$$

where $u_{1Si}$ is the axial displacement of the node i of the micro element; $u_{2Si}$, $u_{3Si}$ are the tangential displacements of the node i of the micro element; $u_{1B}$ is the axial displacement of the macro element node; $u_{2B}$, $u_{3B}$ are the tangential displacements of the macro element node; $u_{4B}$, $u_{5B}$, $u_{6B}$ are the angular displacements of macro element node; $F_{j}$ is the nodal force of macro element in the direction j; *b_i_*, *h_i_* are the distances from the node i of the micro element to the macro element node; $\sigma_{i2,Fj}$, $\sigma_{i3,Fj}$ are the nodal tangential stress of the node i of the micro element caused by $F_{j}$.

In the Equation set (2), the first equation is the axial and rotational constraint equation, which is established by displacement coordination. The last three equations are tangential constraint equations, which are established by energy balance. The aim is to eliminate the limitation of the single constraint relation in the plastic stage and improve the simulation accuracy of the multi-scale model.

#### 3.1.2. Constraint of the Interface in Tangential Direction

**Figure 4:**
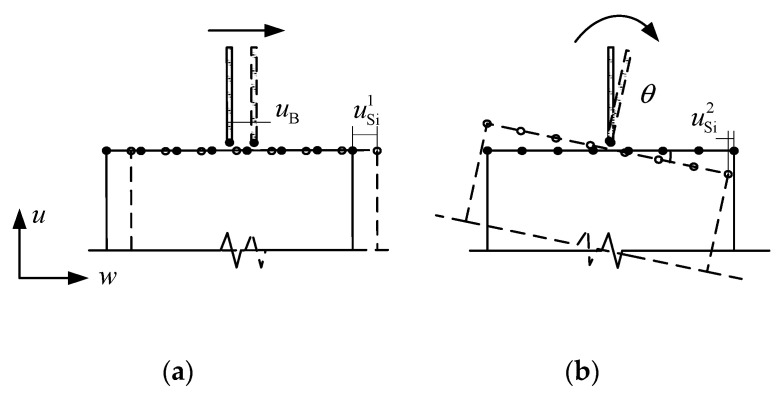
Deformation diagram of displacement coordination. (**a**) Tangential direction; (**b**) Rotational direction.

Based on the multi-point constraint relation of displacement coordination, the tangential deformations of all micro element node at the interface are assumed to be consistent, and the displacement constraint relation of each node is established one by one. The deformation diagrams of displacement coordination are shown in Figure 4, and the constraint equations can be obtained as follows:

(3) $$\begin{array}{l} u_{2Si}-f_{2i}(u_{2B},u_{4B},b_{i},h_{i})=0\\ u_{3Si}-f_{3i}(u_{3B},u_{4B},b_{i},h_{i})=0 \end{array}$$

In the equations, tangential displacements ($u_{2Si}$, $u_{3Si}$) of the micro element node are calculated from $u_{2B}$, $u_{3B}$ and $u_{4B}$. There is no coupling relation between the degrees of freedom of different nodes of micro elements. Under this condition, when there is no nonzero tangential displacement or rotational displacement of macro element node, the tangential displacement of each node of micro elements along the interface is zero. It leads to the problem of over-constraint in tangential direction at the interface under the axial compression load.

**Figure 5:**
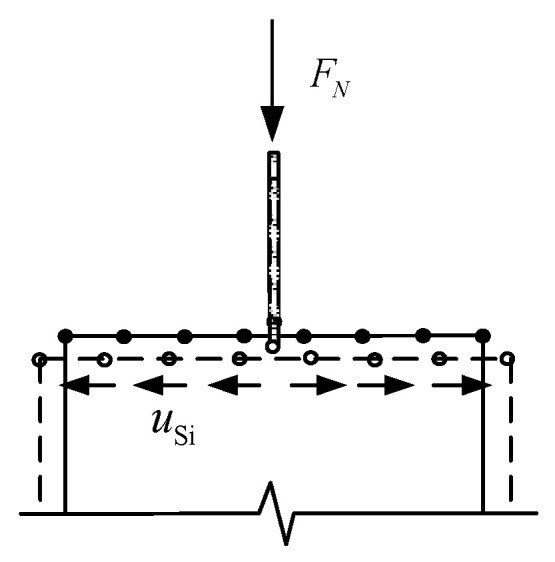
Tangential deformation diagram of CMPC under the axial compression.

In the CMPC equation set, the displacement constraint equation in the tangential direction of the interface nodes can be obtained after the stress is eliminated by substituting the formula for the shear stress distribution:

(4) $$u_{B}=f_{2}(u_{S1},u_{S2}\cdots u_{\mathrm{Sn}})$$

where, the tangential displacements of each micro element node have a coupling relation with each other. When the tangential displacement of macro element node is zero, it can generate the relative displacements among micro element nodes and satisfy the constraint equation. The tangential deformation diagram of CMPC under the axial compression is shown in Figure 5.

**Figure 6:**
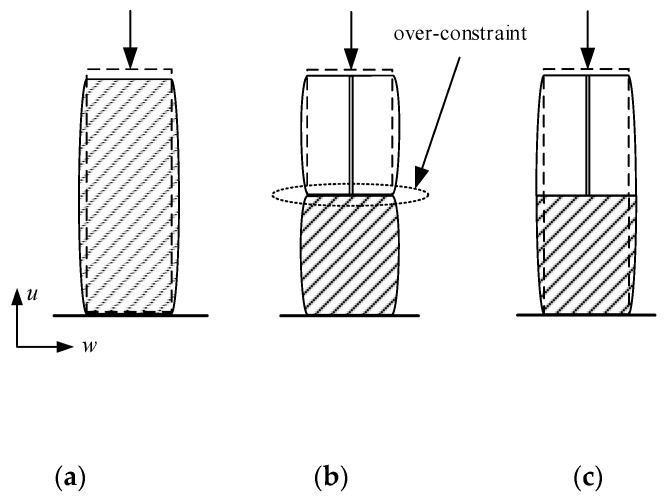
Interface deformation under axial compression. (**a**) Micro element model; (**b**) Multi-scale model of displacement coordination; (**c**) Multi-scale model of CPMC.

The interface deformation under axial compression at the plasticity stage of the multi-scale model is shown in Figure 6. According to the Poisson ratio of UHPFRC, the uniform longitudinal stress causes the transverse strain of the section. And there is obvious transverse expansion deformation in the middle of the micro element model. The micro element nodes at the interface of displacement coordination model only produce vertical displacement with the macro element node with no tangential displacement, which is over-constraint compared with the micro model. The CMPC equation eliminates the over-constraint in tangential direction at the interface and conforms to the deformation relation of the interface nodes under the actual stress state.

#### 3.1.3. Constraint of the Interface in Rotational Direction

The multi-point constraint relation based on energy balance is established by the virtual work principle. It is assumed that the nodal force of the macro element and the nodal forces of the micro elements do equal work at the interface in the rotational direction. The equation is as follows:

(5) $$\begin{array}{l} F_{5}u_{5B}=\int_{A}\sigma_{1i,F5}u_{1Si}dA\\ F_{6}u_{6B}=\int_{A}\sigma_{1i,F6}u_{1Si}dA \end{array}$$

By substituting the formula for stress distribution under bending moment, the displacement constraint equation in rotational direction of the interface nodes can be obtained. Its precision depends on the rationality of the stress distribution of the stress formula.

**Figure 7:**
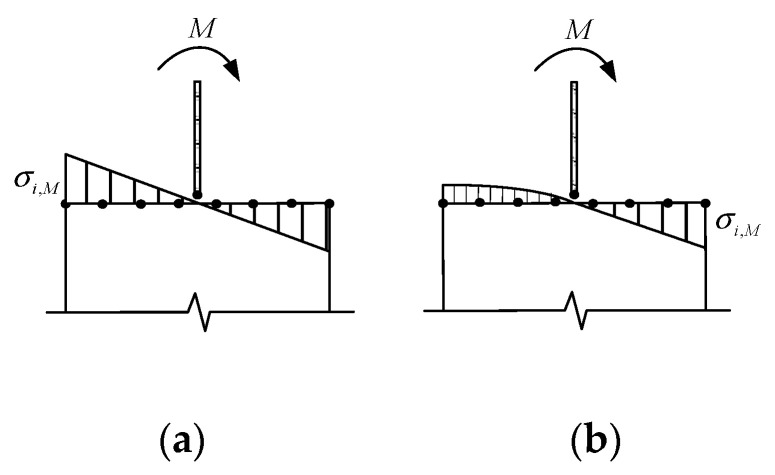
Stress distribution under bending moment. (**a**) Linear-elastic stage; (**b**) Strain hardening stage.

The normal stress distribution of UHPFRC section under bending moment is shown in Figure 7. As in other studies [33,47], the stress distribution of UHPFRC section has underwent different stages. The first stage is the linear-elastic stage, in which the fiber and matrix show elasticity and the stress distribution is linear. With the increase of load, due to the strong bond between the high strength steel fiber and the matrix, the macro crack begins to expand slowly. The strain hardening phenomenon occurred is different from that of NSC, and the tensile stress is nonlinear distributed. This stage is called strain hardening stage and the formula for stress distribution in linear-elastic stage is no longer applicable. If the formula is not updated iteratively, the multi-point constraint equation at the interface based on energy balance will be distorted in the rotational direction in strain hardening stage.

The CMPC method establishes the multi-point constraint equation of the interface in rotational direction based on the displacement coordination. The axial displacement of each node of the micro elements can be obtained through the s constraint equation (see the first equation in Equation set (2)). After entering the strain-hardening stage, this multi-point constraint equation avoids the problem that the formula for stress distribution needs to be updated iteratively. Therefore, the CMPC method proposed in this paper combines the advantages of displacement coordination method and energy balance method. The multi-point constraint equations conform to the transfer relations of displacement and stress between the interface nodes. It can achieve good constraint effect in axial, tangential, and rotational directions. It is applicable to the analysis of UHPFRC components under complex loads.

### 3.2. CMPC Equations of Multi-Scale Connection of Beam-Solid Element

**Figure 8:**
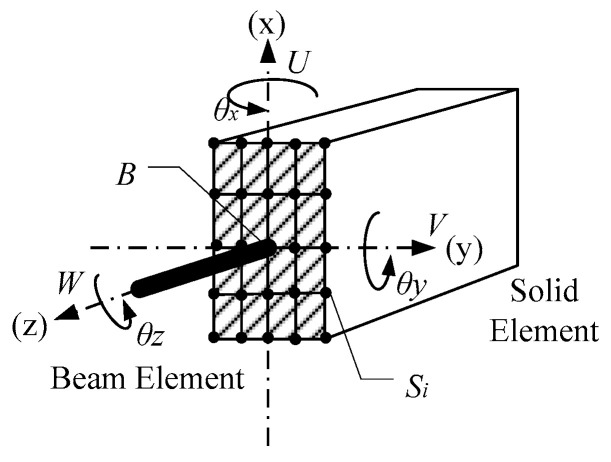
Multi-scale connection of beam - solid element.

According to Equation set (2), displacement vector $\begin{array}{cccccc} {}[u_{B} & v_{B} & w_{B} & \theta_{x} & \theta_{y} & \theta_{z} \end{array}]$ of beam element and displacement vector $\left[\begin{array}{ccc} u_{Si} & v_{Si} & w_{Si} \end{array}\right]$ of solid element are substituted, and the multi-point constraint equations can be expressed as:

(6) $$\left\{ \begin{array}{l} w_{Si}-f_{i}\left(w_{B},\theta_{x},\theta_{y},R_{xi},R_{yi}\right)=0\\ F_{x}u_{B}=\int_{A}\tau_{xi}u_{Si}dA\\ F_{y}v_{B}=\int_{A}\tau_{yi}v_{Si}dA\\ T\theta_{z}=\int_{A}(\tau_{xi}u_{Si}+\tau_{yi}v_{Si})dA \end{array}\right.$$

where $R_{xi}$, $R_{yi}$ are the distances between the node i of the solid element and the beam element node at the interface in the x and y direction, respectively; $F_{x}$, $F_{y}$ are the shear forces acting on the beam element node in the x and y direction, respectively; T is the torque acting on the beam element node; $\tau_{xi}$, $\tau_{yi}$ are the shear stresses of the node i of the solid element in the x and y direction, respectively. The multi-scale connection of beam - solid element is shown in Figure 8.

In the Equation set (6), the first equation is the constraint equation of axial and rotational displacement, which can be solved according to the displacement coordination. The last three equations are the constraint equations of tangential displacements, which need to be solved by substituting the formula for stress distribution. For example, the formula for shear stress distribution of the rectangular section in the y direction is as follows:

(7) $$\tau_{yi}=\frac{3F_{y}}{2bh}\left(1-\frac{4R_{yi}{}^{2}}{h^{2}}\right)$$

where *b* and *h* are the width and height of the rectangular section, respectively.

After solving the Equation set (6), the following can be obtained:

(8) $$\left\{ \begin{array}{l} w_{Si}=w_{B}+R_{xi}\sin\theta_{y}+R_{yi}\sin\theta_{x}\\ u_{B}=C_{u1}u_{1}+C_{u2}u_{2}+\cdot \cdot \cdot +C_{un}u_{n}\\ v_{B}=C_{v1}v_{1}+C_{v2}v_{2}+\cdot \cdot \cdot +C_{vn}v_{n}\\ \theta_{Z}=(u_{1}R_{y1}+\cdot \cdot \cdot +u_{n}R_{yn})-(v_{1}R_{x1}+\cdot \cdot \cdot +v_{2}R_{x2}) \end{array}\right.$$

where $C_{ui}$, $C_{vi}$ are the influence coefficients of the tangential displacements related to the section size and node position. An example of the Equation set (8) is given in Appendix A.

## 4. Multi-Scale Models of Ultra-High-Performance Steel Fiber-Reinforced Concrete

### 4.1. Multi-Scale Models Built-Up

**Figure 9:**
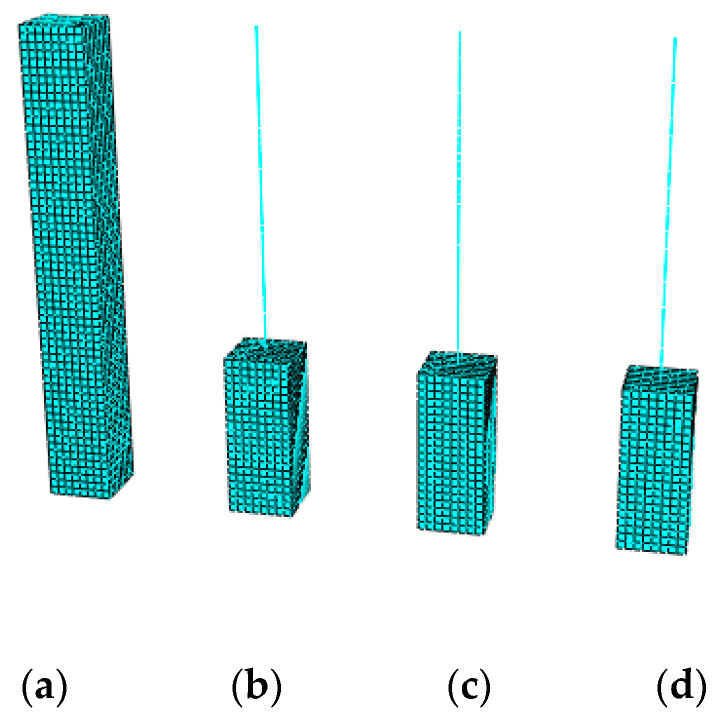
UHPFRC parts of the models. (**a**) Solid element model; (**b**) Displacement coordination model; (**c**) Energy balance model; (**d**) CMPC model.

**Figure 10:**
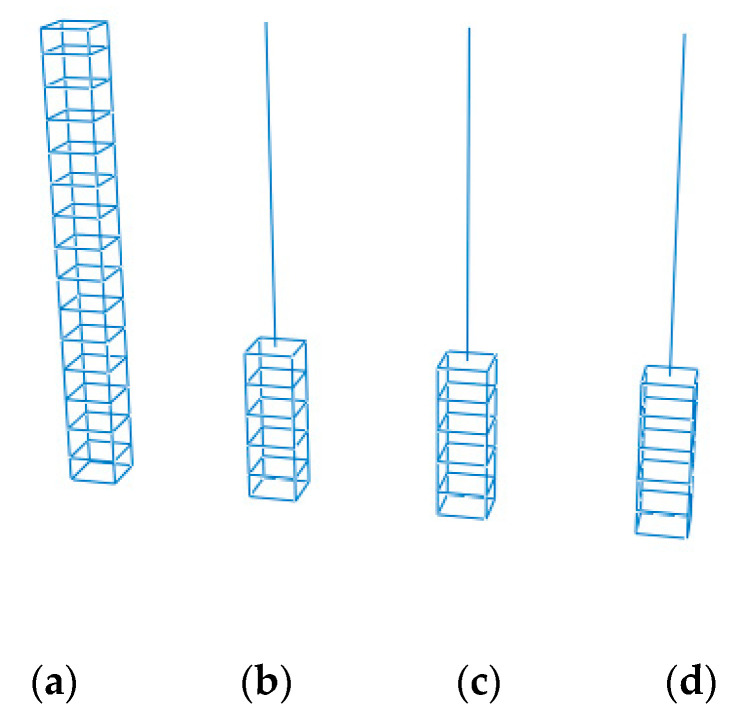
Reinforcement parts of the models. (**a**) Solid element model; (**b**) Displacement coordination model; (**c**) Energy balance model; (**d**) CMPC model.

The CMPC multi-scale modeling strategy with the same parameters selected above is adopted to establish the multi-scale models of reinforced UHPFRC components, as shown in Figure 9 and Figure 10. Where (a) is the solid element model taken as the standard for comparison without experimental results, and (b), (c) and (d) are the multi-scale models of beam-solid element, whose interface connections are established by the displacement coordination method, the energy balance method and the CMPC method (Section 3.2. for the expressions) respectively. The height of the component is 3m, and the section size is 0.4 m × 0.4 m. The multi-scale interface is located at 1/3 height of the component with the height of 3 m. The parameters of the CDP model adopted for the UHPFRC solid elements are shown in Table 1.

In the micro element model, C3D8R elements are used to model UHPFRC with the calibrated CDP model which is the same as that in the second section. The reinforcement is simulated by T3D2 element. In the macro element model, B31 elements are used to simulate UHPFRC and the reinforcement net. The uniaxial stress-strain relationship of the material subroutine (UMAT) of UHPFRC is the same as that of the calibrated CDP model. The section size of the B31 element of the reinforcement net is calculated equivalent to total reinforcement area. The material model parameters of reinforcement are the same as those in Section 2. With a yield strength and ultimate strength of 525 and 625 MPa, respectively. The mesh size of the models is 0.05m. A fixed constraint is set at the bottom of the component and a loading point is set at the top. The number of model elements of the solid element model and the multi-scale model is shown in Table 4. It can be seen that the number of model elements in the multi-scale model is reduced by nearly 2/3 compared with that in the solid element model, which significantly improves the computational efficiency.

### 4.2. Unidirectional Load Cases

#### 4.2.1. Axial Compression Load Case

**Figure 11:**
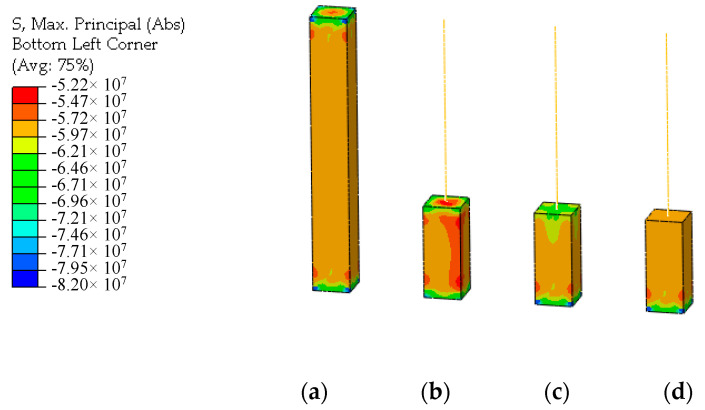
Stress distributions under the axial compression load (unit: Pa). (**a**) Solid element model; (**b**) Displacement coordination model; (**c**) Energy balance model; (**d**) CMPC model.

Under the axial compression load, the stress distributions of UHPFRC of the multi-scale models and the connection interface are shown in Figure 11. By comparison, it can be seen that there is the phenomenon of stress concentration at the connection interface of the displacement coordination model whose stress distribution is different from that of the solid model. The overall stress distribution of the energy balance model is also different from that of the solid model, and the stress distribution at the interface connection is not uniform. The stress distribution obtained by the CMPC method is highly consistent with the solid element model. The constraint effect of the CMPC method is obviously better than that of the single multi-point constraint method.

#### 4.2.2. Bending Load Case

**Figure 12:**
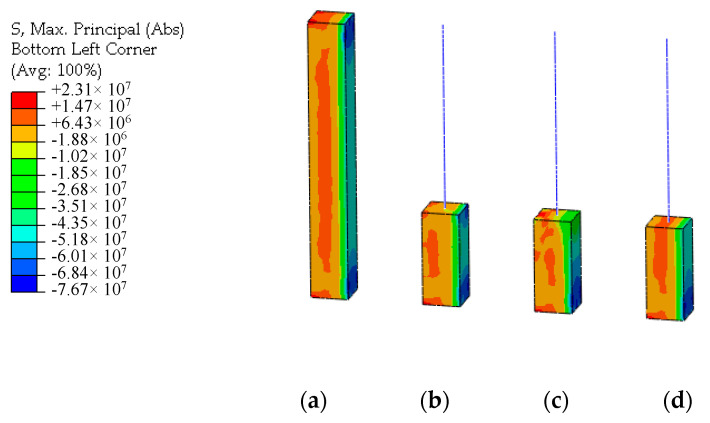
Stress distributions under the bending load (unit: Pa). (**a**) Solid element model; (**b**) Displacement coordination model; (**c**) Energy balance model; (**d**) CMPC model.

**Figure 13:**
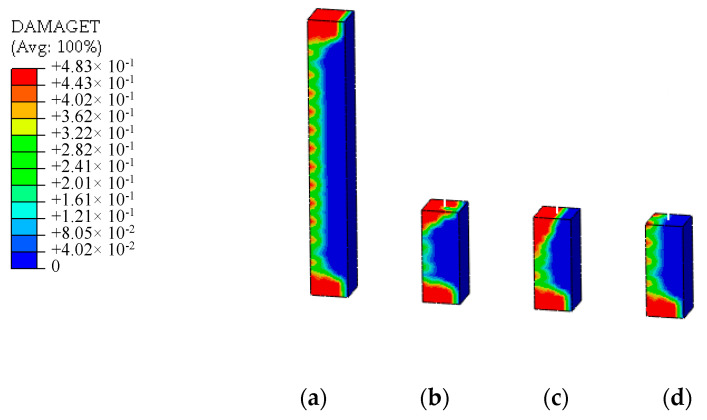
Tensile damage distributions under the bending load (unit: Pa). (**a**) Solid element model; (**b**) Displacement coordination model; (**c**) Energy balance model; (**d**) CMPC model.

Unidirectional concentrated moment loads is applied to the loading point at the top of the models under the bending load case. The stress distribution and tensile damage distribution of UHPFRC of the models are shown in Figure 12 and Figure 13. By comparison, it can be seen that the stress distribution at the connection interface in the plastic stage is nonlinear. The UHPFRC on the tensile side enters the strain hardening stage with tensile damage. Compared with the solid element model, the results of the displacement coordination model and the energy balance model show obvious stress distortion at the connection interface. As UHPFRC on the tension side enters the strain hardening stage, the formula for stress distribution of the energy balance method is no longer applicable. The original constraint equation needs to be balanced by over increasing the strain on the tension side, resulting in the distortion of the damage distribution at the connection interface. Due to the over-constraint in tangential direction mentioned above, the results of the displacement coordination model at the connection interface look distorted. The simulation results of CMPC model are highly consistent with that of the solid element model, and the stress and damage distribution of UHPFRC at the connection interface are simulated accurately.

#### 4.2.3. Shear Load Case

**Figure 14:**
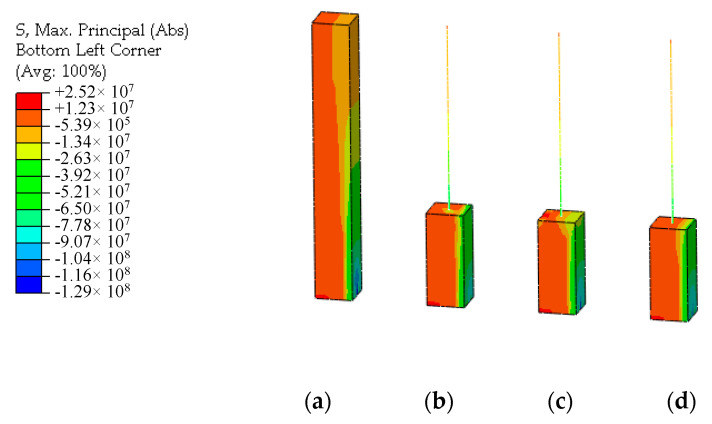
Stress distributions under the shear compression load (unit: Pa). (**a**) Solid element model; (**b**) Displacement coordination model; (**c**) Energy balance model; (**d**) CMPC model.

**Figure 15:**
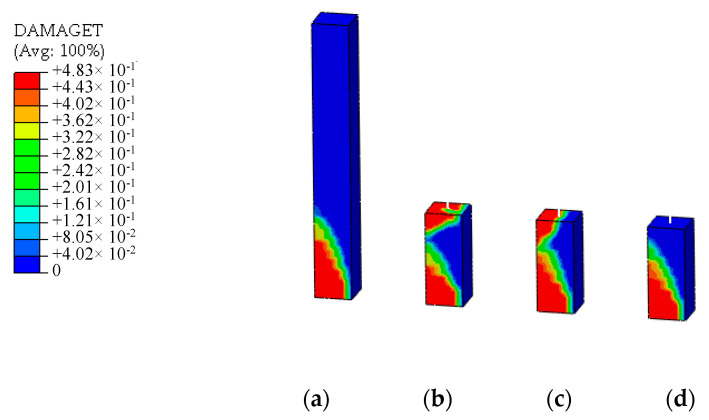
Stress distributions under the shear compression load (unit: Pa). (**a**) Solid element model; (**b**) Displacement coordination model; (**c**) Energy balance model; (**d**) CMPC model.

In shear load case, a concentrated horizontal force is applied to the loading point at the top of the models. The stress distribution and tensile damage distribution of UHPFRC of the models are shown in Figure 14 and Figure 15. It can be seen that after entering the strain hardening stage of UHPFRC, the stress distribution at the connection interface presents nonlinear. The multi-point constraint equation derived from the formula for elastic stress distribution is no longer applicable, and the simulation results of the stress distribution and damage distribution at the connection interface of the energy balance model are inaccurate. At the same time, due to the tangential over-constraint, the stress concentration occurs at the connection interface of the displacement coordination model. The damage distribution is distorted. However, the simulation results of the CMPC model still have good accuracy. The stress and damage distribution of UHPFRC at the connection interface are simulated accurately.

### 4.3. Multidirectional Composite Load Case

Under unidirectional load cases, the multi-scale model of UHPFRC component established according to the proposed multi-scale modeling strategy achieved good accuracy. The performance of this multi-scale model under multidirectional composite load case will be studied below.

**Figure 16:**
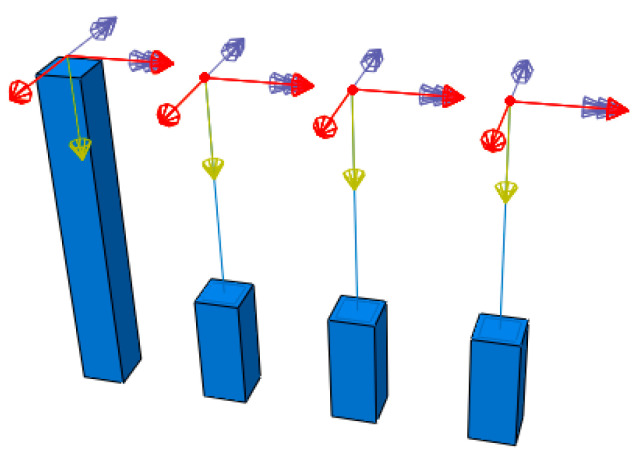
The diagram of multidirectional composite load.

In this load case, the axial compression force, the bidirectional moments and the bidirectional horizontal forces are applied composited at the loading point at the top of the models. The diagram of multidirectional composite load is shown in Figure 16. Where, the red arrow represents the bidirectional horizontal forces, the yellow arrow represents the axial compression force, and the purple double arrow represents the bidirectional moments.

**Figure 17:**
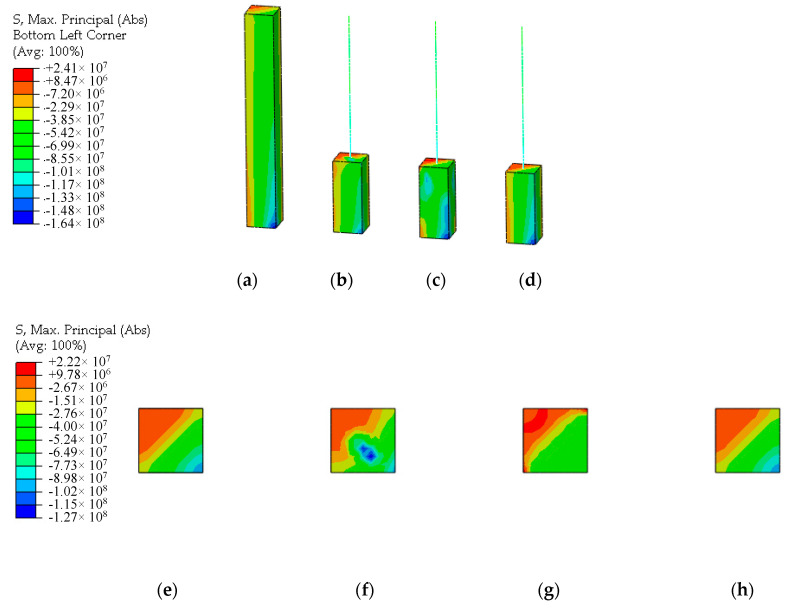
Stress distributions under the multidirectional composite loads case (unit: Pa). (**a**) Solid element model; (**b**) Displacement coordination model; (**c**) Energy balance model; (**d**) CMPC model; (**e**) Interface location of the solid element model; (**f**) Interface of the displacement coordination model; (**g**) Interface of the energy balance model; (**h**) Interface of CMPC model.

Under the multidirectional composite load case, the stress distributions of UHPFRC of the multi-scale models and the connection interface are shown in Figure 17.

Through comparison, it can be seen that the CPMC equations established based on the proposed multi-scale modeling strategy achieve good connection effect under the multidirectional composite load case. The calculation accuracy of the CMPC model for UHPFRC is consistent with that of the solid element model, which is better than the displacement coordination model and the energy balance model. The multi-scale modeling strategy proposed in this study can be effectively applied to the multi-scale finite element analysis of UHPFRC structures with accuracy and efficiency.

## 5. Conclusions

This study proposed a novel multi-scale modeling strategy for ultra-high-performance steel fiber-reinforced concrete (UHPFRC) structures. The main work and conclusions are summarized as follows:

1. The applicability of concrete damage plasticity (CDP) model in finite element software ABAQUS to UHPFRC was verified according to the four-point bending test results of reinforced UHPFRC beams. The simulation results show that the calibrated CDP model used in the multi-scale modeling strategy in this study can accurately and effectively predict the load-displacement curve and plastic damage distribution of UHPFRC components.
2. A novel combined multi-point constraint method was established by the simultaneous equations of the displacement coordination equation and energy balance equation in different directions of the interface. The CMPC method eliminates the problem of the tangential over-constraint of displacement coordination equation at the interface and avoids stress iteration of energy balance equation in the plastic stage. The multi-point constraint equations conform to the transfer relations of displacement and stress between the interface nodes.
3. The expression of the constraint equations of the multi-scale connection of beam-solid element by CMPC method was derived. The multi-scale model of the reinforced UHPFRC component was established in ABAQUS with this expression and the calibrated CDP model. The axial compression load case, bending load case, shear load case, and multidirectional composite load case are analyzed.
4. The simulation results of the multi-scale model under each load case show that the multi-scale model established by the CMPC method can significantly reduce the number of model elements and improve the calculation efficiency. The CMPC models have good simulation accuracy in the analysis of each load case compared with the displacement coordination model and the energy balance model. In the strain-hardening stage of UHPFRC, the CMPC method can still accurately simulate the stress distribution and damage distribution of the connection interface. It can be applied to multi-scale finite element analysis of UHPFRC structures with accuracy and efficiency.

## Figures and Tables

**Table 1:** Parameters of the concrete damage plasticity (CDP) model of ultra-high-performance fiber-reinforced concrete (UHPFRC).

Model Name	Young’s Modulus	Compressive Strength	Tension Yield Stress	Tension Peak Stress
CDP	36.3 GPa	140 MPa	4.6 MPa	5.8 MPa
Dilation angle	Eccentricity	*f*_b0_/*f*_c0_	*k*_c_	Viscosity Parameter
30	0.1	1.05	2/3	0.005

**Table 2:** Specimens details. [34].

Specimen Name	Cross Section (mm)	Effective Span (mm)	Loading Condition	Length of the Midspan without Stirrup (mm)
B25-1 & B25-2	250 × 250	3250	Four point bending	500
Top rebar diameter (mm)	Top rebar number	Bottom rebar diameter (mm)	Bottom rebar number	Stirrup diameter and spacing (mm)
10	2	20	3	D10@90

**Table 3:** Comparison of the FE model results with test results.

Specimen Name	Peak Load (kN)		Relative Error	Peak Displacement (mm)		Relative Error
	FE Model	Test		FE Model	Test	
B25-1	174	172	1.16%	40	59	−32%
B25-2	174	167	4.19%	40	39	2.56%

**Table 4:** Number of model elements.

Model	Element Type			Total
	C3D8R	T3D2	B31	
Solid element model	3840	616	0	4456
Multi-scale model	1280	220	80	1580

**Figure A1:**
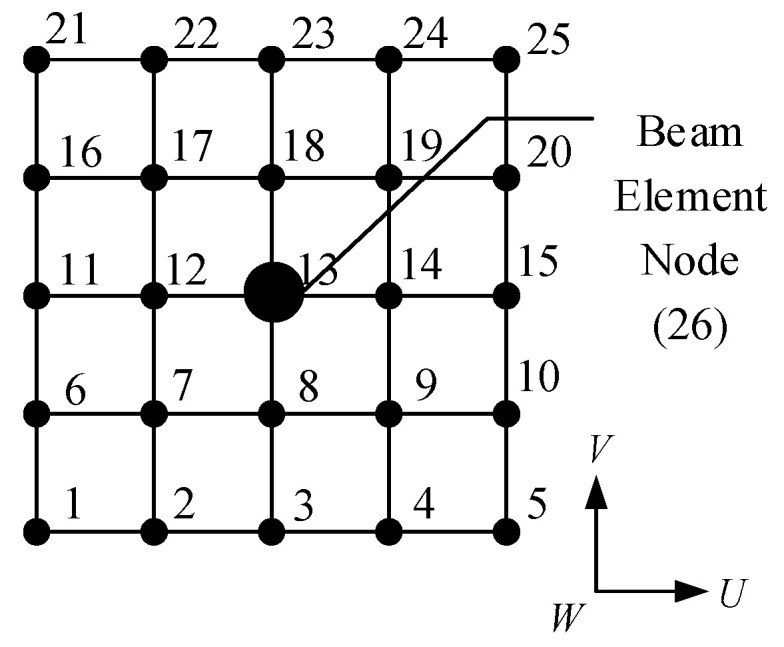
An example of the multi-scale connection interface.

## Appendix A

An example of the multi-scale connection interface is established with section size 0.4 m × 0.4 m and mesh size 0.1 m shown in Figure A1, where the solid element node number on the connection interface is 1–25, and the beam element node number is 26.

The Equation set (8) of the multi-scale connection interface shown in Figure A1 can be obtained as follow. The multi-scale connection of CMPC model can be established by coding the ‘*EQUATION’ keyword commands to input the constrained Equation set (8) into the ‘*.inp’ file of ABAQUS. Similarly, this strategy can be implemented in the other FEA software by setting the multi-point constraint equations.

(A1) $$\left\{ \begin{array}{l} w_{Si}=w_{B}+R_{xi}\sin\theta_{y}+R_{yi}\sin\theta_{x}\\ 1024u_{26}=[7(u_{25}+u_{1}+u_{5}+u_{21})+14(u_{20}+u_{15}+u_{10}+u_{16}+u_{11}+u_{6})+34(u_{24}+u_{22}+u_{4}+u_{2})+\\ \;\;\;\;68(u_{19}+u_{14}+u_{9}+u_{17}+u_{12}+u_{7})+46(u_{23}+u_{3})+92(u_{8}+u_{13}+u_{18})]\\ 1024v_{26}=[7(v_{25}+v_{1}+v_{5}+v_{21})+14(v_{24}+v_{22}+v_{4}+v_{2}+v_{23}+v_{3})+34(v_{20}+v_{16}+v_{10}+v_{6})+\\ \;\;\;\;68(v_{19}+v_{17}+v_{9}+v_{7}+v_{18}+v_{8})+46(v_{15}+v_{11})+92(v_{14}+v_{12}+v_{13})]\\ 10\theta_{z26}=2(u_{1}+u_{2}+u_{3}+u_{4}+u_{5}+v_{5}+v_{10}+v_{15}+v_{20}+v_{25})+\\ \;\;\;\;\;2(-u_{21}-u_{22}-u_{23}-u_{24}-u_{25}-v_{1}-v_{6}-v_{11}-v_{16}-v_{21})+\\ \;\;\;\;\;(u_{6}+u_{7}+u_{8}+u_{9}+u_{10}+v_{4}+v_{9}+v_{14}+v_{19}+v_{24})+\\ \;\;\;\;\;(-u_{16}-u_{17}-u_{18}-u_{19}-u_{20}-v_{2}-v_{7}-v_{12}-v_{17}-v_{22})\\ w_{1}=w_{26}-0.2\sin\theta_{x26}+0.2\sin\theta_{y26}\\ w_{2}=w_{26}-0.2\sin\theta_{x26}+0.1\sin\theta_{y26}\\ w_{3}=w_{26}-0.2\sin\theta_{x26}\\ w_{4}=w_{26}-0.2\sin\theta_{x26}-0.1\sin\theta_{y26}\\ w_{5}=w_{26}-0.2\sin\theta_{x26}-0.2\sin\theta_{y26}\\ w_{6}=w_{26}-0.1\sin\theta_{x26}+0.2\sin\theta_{y26}\\ w_{7}=w_{26}-0.1\sin\theta_{x26}+0.1\sin\theta_{y26}\\ w_{8}=w_{26}-0.1\sin\theta_{x26}\\ w_{4}=w_{26}-0.1\sin\theta_{x26}-0.1\sin\theta_{y26}\\ w_{10}=w_{26}-0.1\sin\theta_{x26}-0.2\sin\theta_{y26}\\ w_{11}=w_{26}+0.2\sin\theta_{y26}\\ w_{12}=w_{26}+0.1\sin\theta_{y26}\\ w_{13}=w_{26}\\ w_{14}=w_{26}-0.1\sin\theta_{y26}\\ w_{15}=w_{26}-0.2\sin\theta_{y26}\\ w_{16}=w_{26}+0.1\sin\theta_{x26}+0.2\sin\theta_{y26}\\ w_{17}=w_{26}+0.1\sin\theta_{x26}+0.1\sin\theta_{y26}\\ w_{18}=w_{26}+0.1\sin\theta_{x26}\\ w_{19}=w_{26}+0.1\sin\theta_{x26}-0.1\sin\theta_{y26}\\ w_{20}=w_{26}+0.1\sin\theta_{x26}-0.2\sin\theta_{y26}\\ w_{21}=w_{26}+0.2\sin\theta_{x26}+0.2\sin\theta_{y26}\\ w_{22}=w_{26}+0.2\sin\theta_{x26}+0.1\sin\theta_{y26}\\ w_{23}=w_{26}+0.2\sin\theta_{x26}\\ w_{24}=w_{26}+0.2\sin\theta_{x26}-0.1\sin\theta_{y26}\\ w_{25}=w_{26}+0.2\sin\theta_{x26}-0.2\sin\theta_{y26} \end{array}\right.$$
